# Iodonium-based pro-adhesive layers for robust adhesion of PEDOT:PSS to surfaces

**DOI:** 10.1080/14686996.2024.2338786

**Published:** 2024-04-04

**Authors:** Szymon Smołka, Taral Patel, Sandra Pluczyk-Małek, Roman Turczyn, Katarzyna Krukiewicz

**Affiliations:** a Department of Physical Chemistry and Technology of Polymers, Silesian University of Technology, Gliwice, Poland; b Joint Doctoral School, Silesian University of Technology, Gliwice, Poland; c Centre for Organic and Nanohybrid Electronics, Silesian University of Technology, Gliwice, Poland

**Keywords:** Adhesion, charge storage capacity, charge transfer, electrografting, iodonium salts, PEDOT:PSS

## Abstract

Electrochemical grafting of organic molecules to metal surfaces has been well-known as an efficient tool enabling tailored modification of surface at the nanoscale. Among many compounds with the ability to undergo the process of electrografting, iodonium salts belong to less frequently used, especially when compared with the most popular diazonium salts. Meanwhile, due to their increased stability, iodonium salts may be used in situations where the use of diazonium salts is constrained. The aim of this study was to examine the effect of the electrochemical reduction of iodonium salts on the physicochemical properties of Pt electrodes, and the possibility to form pro-adhesive layers facilitating further functionalization purposes. Consequently, we have selected four commercially available iodonium salts (diphenyliodonium chloride, bis(4-tertbutylphenyl)iodonium hexafluorophosphate, (4-nitrophenyl)(2,4,6-trimethylphenyl)iodonium triflate, bis(4-methylphenyl)iodonium hexafluorophosphate), and attached them to the surface of Pt electrodes by means of an electrochemical reduction process. As-formed layers were then extensively characterized in terms of wettability, roughness and charge transfer properties, and used as pro-adhesive coatings prior to the deposition of poly(3,4-ethylenedioxythiophene):poly(styrene sulfonate), PEDOT:PSS. Due to the increase in hydrophilicity and roughness, modified electrodes increased the stability of PEDOT:PSS coating while maintaining its high capacitance.

## IMPACT STATEMENT

Adhesion and charge transfer between PEDOT:PSS and the surface of the electrode are significantly improved by a simple electrode modification strategy using the electrochemical grafting of commercially available iodonium salts.

## Graphical abstract

**Figure:**
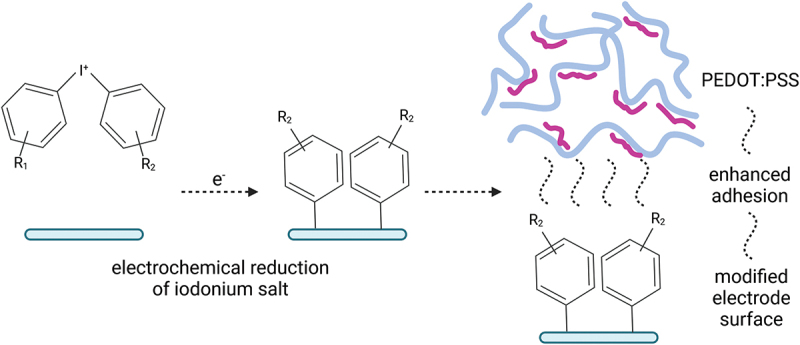

## 1. Introduction

In the realm of surface functionalization, diazonium salts have emerged as remarkable and versatile tools enabling tailored modification of surface at the nanoscale [1,2]. The ability of diazonium salts to functionalize a diverse array of substrates has ignited a substantial interest, fueling their adoption in various fields [3]. These compounds, possessing an aryl-diazonium group, exhibit inherent electroactivity that renders them suited for direct attachment to surfaces through a process of electrochemical reduction. By capitalizing on the electrochemical properties of diazonium salts, researchers have unlocked an innovative approach to tailor surface characteristics, in terms of wettability, roughness, as well as chemical and biological activity [4]. The versatility of diazonium salts is demonstrated through a range of applications across diverse fields, including the development of corrosion-resistant coatings, enhancement of biocompatibility of biomedical devices, and modification of conductive materials for advanced electronics [5–7]. The electrodeposition of diazonium salts involves the controlled reduction of the aryl-diazonium group, leading to its covalent bonding with the substrate [8]. Exploring the intricate mechanism underlying this reaction unveils fundamental principles driving the selectivity and efficiency of diazonium salt-based surface functionalization. A key to the success of diazonium salt-based surface modification is the careful optimization of reaction conditions [4]. Despite their remarkable versatility, diazonium salts do possess limitations, including low solubility in aqueous media, the need to assure substrate compatibility and challenges in achieving uniform coverage over large surface areas [9]. To expand the pool of plausible grafting agents, a wide variety of diazonium salt alternatives that possess stable precursors, including alkyl and aryl iodides [10], sulfonium [11] and iodonium salts [12], have been used so far. Amongst them, iodonium salts have emerged as a potentially preferred choice due to their increased stability, particularly when compared with diazonium salts [13].

Iodonium salts, which are characterized by the presence of an aryl-iodonium group, offer a unique set of attributes that position them as superior agents for surface modification. The inherent reactivity of iodine and the electroactivity of the iodonium moiety synergize to enable efficient and controlled surface functionalization [14]. This synergy allows for the direct and site-specific attachment of functional groups, facilitating the formation of surfaces with finely tuned chemical, physical, and electrical properties [15]. The distinct reactivity of iodonium salts opens the door to a wide array of applications, spanning fields from materials science to biomedical engineering [16]. One of the foremost advantages of iodonium salts lies in their increased stability compared to diazonium salts [17]. Iodonium salts exhibit greater tolerance to a variety of reaction conditions, including temperature and pH fluctuations [18]. This resilience enables researchers to explore a broader range of substrates and experimental environments without compromising the success of the modification process. Furthermore, the stability of iodonium salts contributes to an enhanced shelf life, ensuring consistent and reproducible outcomes over extended periods [19].

Both diazonium and iodonium salts are known to serve as ‘molecular glues’; electrodeposition of these salts yields the formation of organic layers with pro-adhesive properties, able to serve as coupling agents for further functionalization purposes [7]. One of the most notable applications of electrodeposited diazonium salts is their use as an interfacial layer between the surface of the electrode and a conducting polymer layer, resulting in the formation of a conducting coating characterized by a better affinity to the electrode and higher adhesion force [20]. Poly(3,4-ethylenedioxythiophene):poly(styrene sulfonate), PEDOT:PSS is a conducting polymer which, due to its high electrical conductivity and thermal stability, is widely used in numerous applications, including photovoltaics [21,22], energy storage devices [23], bioelectronics [24], biosensors [25,26], wearable devices [27,28] and biofuel cells [29]. PEDOT:PSS can be also used as a coating for biomedical devices, such as neural implants [30]. Despite the promising properties of PEDOT:PSS, however, there are still several key problems to solve before this polymer can be widely used in biomedicine. One of the major limitations of PEDOT:PSS is its poor adhesion, which causes the polymer to swell and detach from the electrode’s surface. Several methods have been proposed in the literature to solve this problem, such as the use of a layer of hydrophilic adhesive strongly adhering to the substrate through which a conducting polymer can penetrate [31], by attaching PEDOT to a previously modified substrate using diazonium salts [32], by plasma treatment [32], or by solvent treatment, which also increases the conductivity of the polymer [33].

In our previous work, we found that the modification of a surface of an electrode with a post-diazonium layer has a positive effect on the adhesion of PEDOT:PSS [20]. In this study, we aimed to examine whether iodonium salts can also facilitate the adhesion of PEDOT:PSS, and whether they could outperform diazonium salts, while eliminating their disadvantages. Consequently, we selected four commercially available iodonium salts, and attached them to the surface of a Pt electrode by means of an electrochemical reduction process. As-formed layers were then extensively characterized, and used as pro-adhesive coatings prior to the deposition of PEDOT:PSS through a drop casting technique. The functionality of deposited PEDOT:PSS was evaluated in terms of its electrical properties, and its stability was estimated after 30 days of ageing with the use of UV-Vis spectroscopy. Previously, few papers describing the influence of iodonium salts on polymer adhesion were published [34–37], but none of them concerned surface modification through grafting of those salts. Iodonium salts were used as photoinitiators in ternary system, where their addition led to better adhesion and mechanical properties of biomedical resin [35,36] or adhesives used in dental application [34,37]. To the best of our knowledge, there are no previous investigation on the effect of grafting iodonium salts on the adhesion of polymers, in particular PEDOT:PSS.

## 2. Materials and methods

### 2.1. Reagents

Diphenyliodonium chloride, bis(4-tertbutylphenyl)iodonium hexafluorophosphate, (4-nitrophenyl)(2,4,6-trimethylphenyl)iodonium triflate, bis(4-methylphenyl)iodonium hexafluorophosphate, tetrabutylammonium hexafluorophosphate (*t*Bu_4_NPF_6_) were obtained from Sigma-Aldrich. Phosphate-buffered saline (PBS) tablets (Fisher Bioreagents) were dissolved in deionized water to obtain a solution containing 0.01 M phosphate buffer, 0.0027 M KCl and 0.127 M NaCl. Acetonitrile (ACN, 99.9%), ferrocene, KNO_3_ were purchased from Sigma-Aldrich. *t*Bu_4_NPF_6_ was vacuum dried before use. Aqueous dispersion of PEDOT:PSS was purchased from either Ossila (PH1000) or Sigma Aldrich (1.1%), as indicated. Other reagents were used as received.

### 2.2. Electrochemical studies

Electrochemical reduction of bis(4-tertbutylphenyl)iodonium hexafluorophosphate (Salt A), (4-nitrophenyl)(2,4,6-trimethylphenyl)iodonium triflate (Salt B), diphenyliodonium chloride (Salt C) and bis(4-methylphenyl)iodonium hexafluorophosphate (Salt D), was carried out using CHI 660 potentiostat (CH Instruments, Texas, U.S.A.). A conventional three electrode setup was used consisting of a platinum disc or thermanox coverslips (Thermo Scientific, MA, U.S.A., Nunc TM, 22 mm x 60 mm) coated with a sputter-coated platinum layer (Q150R Quorum technologies, UK) as a working electrode, Ag/AgCl (3 M KCl) (eDAQ, Australia) reference electrode (for aqueous solutions) or Ag wire pseudoreference electrode (for organic solutions) and a platinum auxiliary electrode. The potential of the pseudoreference electrode was monitored using ferrocene (Fc). Salts A, B and D (3 mM) were dissolved in ACN containing 0.1 M *t*Bu_4_NPF_6_ as a supporting electrolyte. Salt C (3 mM) was dissolved in deionized water containing 0.1 M KNO_3_ as a supporting electrolyte. The deposition process was carried out by the means of cyclic voltammetry within the potential ranges: from −0.4 V to −1.6 V vs. Fc/Fc^+^ (salt A), from 0 V to −0.8 V vs. Fc/Fc^+^ (salt B), from −0.1 V to −0.7 V vs. Fc/Fc^+^ (salt C) and from −0.5 V to −1.5 V vs. Fc/Fc^+^ (salt D), at the scan rate of 50 mV/s.

Following the electrochemical reduction, modified electrodes were analyzed by means of an electrochemical impedance spectroscopy (EIS) employing CHI 660c potentiostat. The measurements were conducted in a PBS solution in the frequency range from 0.1 Hz to 100 kHz, and AC amplitude of 50 mV. The results were shown in the form of Nyquist and Bode plots. Data fitting was performed with the use of EIS spectrum analyzer software with a Nelder-Mead simplex algorithm [38].

Capacitance, **C** (µF) of a constant phase element (CPE) was calculated by using the following equation [20]

(1) $$C=\frac{{\left(Q\cdot R_{\mathrm{CT}}\right)}^{\left(\frac{1}{n}\right)}}{R_{\mathrm{CT}}}$$

where **Q** is the CPE parameter, **R**_**CT**_ is the charge transfer resistance (Ω) and **n** is the CPE exponential parameter.

All experiments were performed at room temperature and pressure (20°C, 1 atm), in the absence of purging preceding to the measurement.

### 2.3. Electrogravimetric investigation of deposition process

Electrogravimetric investigations were performed *via* a time resolved electrochemical quartz crystal microbalance (EQCM, CHI 400C, CH Instruments, Texas, U.S.A) using CHI 125 gold crystal electrodes. The deposition process was carried out by the means of cyclic voltammetry within the potential ranges: from −0.4 V to −1.6 V (salt A), from −0.4 V to −1.2 V (salt B), from −0.1 V to −0.7 V (salt C) and from −0.4 V to −1.6 V (salt D), at the scan rate of 50 mV/s. Deposited masses were calculated by using a Sauerbrey equation [39]:

(2) $$\Delta f=-\frac{2f_{0}^{2}}{A\sqrt{\rho_{q}\mu_{q}}}\Delta m$$

where: $\rho_{q}$ means a density of quartz (2.648 g/cm^3^), $\mu_{q}$ is a shear modulus of quartz (2.947∙10^11^ g cm/s^2^), *A* is an electrode surface (0.205 cm^2^), $f_{0}$ is a resonant frequency of the fundamental mode (Hz), Δm is a mass change (g). For ${\text{\textbackslash{}i}f}_{0}$ = 7.995 MHz, the mass change correlated to a change of 1 Hz of frequency is equal to 1.4 ng.

To eliminate the risk of overestimation of a deposited resulting from the electroreduction of Salt C, arising from the fact that one of the side products of this process (iodobenzene) is insoluble in aqueous medium, Δm acquired for Salt C was multiplied by 0.274, since the mass of benzene ring which attaches to the surface is equal to 27.4% of molecular mass of Salt C.

### 2.4. Surface characteristics

Morphology of electrodeposited layers was examined with the use of an optical profilometer (Profilm 3D, Filmetrics, CA, U.S.A.) and an atomic force microscope (CoreAFM Nanosurf, Switzerland) with the application of a phase contrast (tapping mode) using Tapping Mode HQ:NSC15/AlBS AFM Probe (MikroMasch, CA, U.S.A.). Surface roughness was obtained according to ISO 25,178 using an average surface parameter (S_a_). Wettability of the samples was analyzed by means of an optical goniometer (OCA15 Dataphysisc, CA, U.S.A.) at room temperature (20°C) using deionized water.

### 2.5. Deposition of PEDOT:PSS coating

PEDOT:PSS coatings were deposited on the surface of thermanox/platinum electrodes, previously modified with electroreduced iodonium salts. Substrates were covered with 100 µl of PEDOT:PSS (Ossila) using a drop-casting method and dried at 60°C for 30 minutes.

### 2.6. Electrical properties of PEDOT:PSS

The effect of the presence of an organic layer on electrical properties of PEDOT:PSS (Ossila) was examined with the use of cyclic voltammetry and electrochemical impedance spectroscopy. Pt/Salt/PEDOT:PSS slides were used as working electrodes immersed in PBS, with a Ag/AgCl electrode as a reference, and Pt as a counter electrode. CV scans were performed with different scan rates (200 mV/s, 100 mV/s, 50 mV/s, 20 mV/s, 10 mV/s) at the potential range from −0.5 V to 0.5 V vs. Ag/AgCl. Charge storage capacity, CSC (mC/cm^2^) was calculated by using the following equation [40]:

(3) $$\mathrm{CSC}=\;\frac{1}{A\cdot v}\int_{V_{1}}^{V_{2}}I\left(V\right)\mathrm{dV}$$

where: ***A*** is electrode area (0.283 cm^2^), v is a scan rate (V/s), V is the potential (V) and ***I*** is current (A). ***V***_*1*_ and ***V***_*2*_ represent the potential range of a single scan.

Electrochemical impedance spectra were collected in PBS solution (2 ml) in the frequency range from 0.1 Hz to 100 kHz and an AC amplitude of 50 mV. The results were shown in the form of Nyquist and Bode plots. Data fitting was performed with the use of EIS spectrum analyzer software with a Nelder-Mead simplex algorithm [38].

### 2.7. Stability studies

To investigate the effect of iodonium treatment on the adhesion of PEDOT:PSS (Ossila), an ageing experiment was carried out, in which the modified electrodes were immersed in equal volumes of PBS (2 ml).The bottles were tightly closed to limit the evaporation of the solution and left for 30 days in a dark place. After this time, the solutions were examined using UV-Vis spectroscopy (HO 8453, Hewlett Packard, CA, U.S.A.). CV curves were collected before and after an ageing test, and the corresponding CSC values were calculated according to Equation (3).

The adhesion of PEDOT:PSS (Sigma Aldrich) to modified electrodes was tested by an adhesive tape test, as well. Thermanox/platinum electrodes were modified by studied salts and covered by 200 µl of 1.1% PEDOT:PSS (Sigma Aldrich) water dispersion using a drop casting method. The electrodes were dried at 60°C for 30 min. After that, a commercially available adhesive tape was applied on a half of the electrode’s surfaces, and after 30 s it was removed at a constant force while maintaining 90° angle, the same for each sample.

## 3. Results and discussion

### 3.1. Electrodeposition of iodonium salts

**Figure 1.**
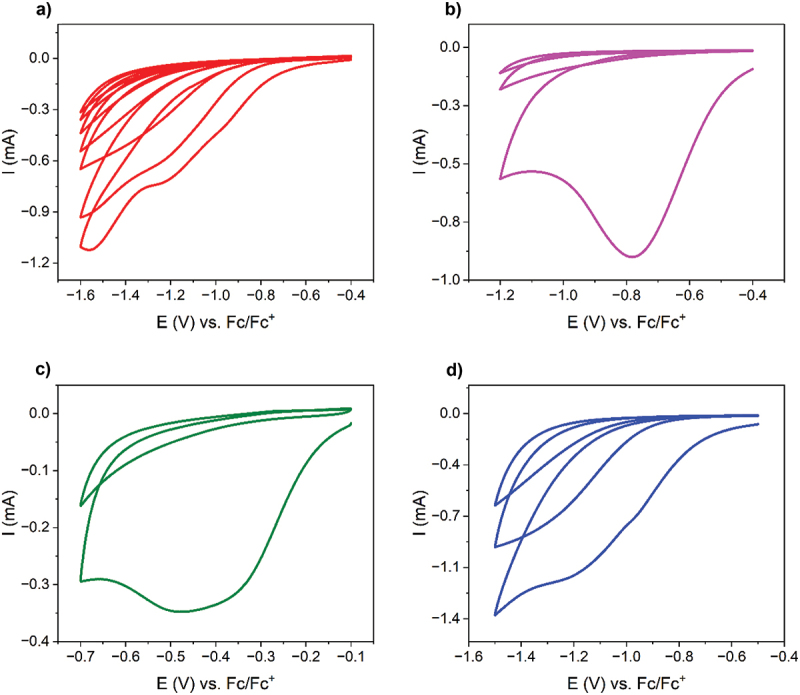
Cyclic voltammograms of the process of electroreduction of 3 mM solutions of iodonium salts: (a) bis(4-tertbutylphenyl) iodonium hexafluorophosphate (**Salt A**), (b) (4-nitrophenyl)(2,4,6-trimethylphenyl)iodonium triflate (**Salt B**), (c) diphenyliodonium chloride (**Salt C**) and (d) bis(4-methylphenyl)iodonium hexafluorophosphate **(Salt D**).

Four iodonium salts with varying functional groups were electrochemically reduced onto the surface of a platinum working electrode *via* cyclic voltammetry, and the CV curves are presented in Figure 1. Salt A, Salt B, and Salt D were dissolved in 0.1 M *t*Bu_4_NPF_6_/ACN to ensure the electroreduction process to take place. On the other hand, water-solubility of Salt C allowed the process of electroreduction to be performed in the 0.1 M KNO_3_/water system without the use of organic solvents, which greatly expanded its applicability and diminished costs.

Different potential ranges have been selected for Salts A-D to assure their efficient electrodeposition, which occurred at the reduction potential of −1.2 V vs. Fc/Fc^+^ (Salt A), −0.8 V vs. Fc/Fc^+^ (Salt B), −0.4 V vs. Fc/Fc^+^ (Salt C), and −1.1 V vs. Fc/Fc^+^ (Salt D). Clearly, the least negative potential of electroreduction process was observed for Salt C, and this difference should be related to the nature of the solvent (water) and the presence of an unsubstituted aromatic ring. For Salt B and Salt C, the process of electroreduction was completed within 1 CV cycle, since there was no reduction peak observed in the next CV cycles. Such behaviour is expected to occur with the progress in electrode passivation as an outcome of the formation of an organic layer on the electrode surface blocking further electrochemical reaction [36]. For Salt A and Salt D, the reduction peaks were still noted in the subsequent CV cycles, however, slightly shifted to more negative potentials and characterized with less negative currents. It is possible that for these salts the aryl radicals formed as a result of electrochemical reduction of iodonium salts were further reduced yielding aryl anions, as observed previously [15,41].

The reduction potentials of iodonium salts are approx. 0.5 V more negative than for their diazonium salt-based analogues, due to their higher stability when compared with diazonium salts [15]. Iodine atom has a larger covalent radius (139 pm) than nitrogen (70 pm) and is characterized by a more diffuse electron density resulting in a more stable positive charge on the iodine atom, which makes the I-Ar bond less prone to cleavage. Even though this makes the electrografting process more challenging, since more negative potentials are required to initiate the process of deposition, higher stability of iodonium salts increases safety in their use by avoiding the possibility of rapid decomposition responsible for the explosive character of some diazonium salts.

**Figure 2.**
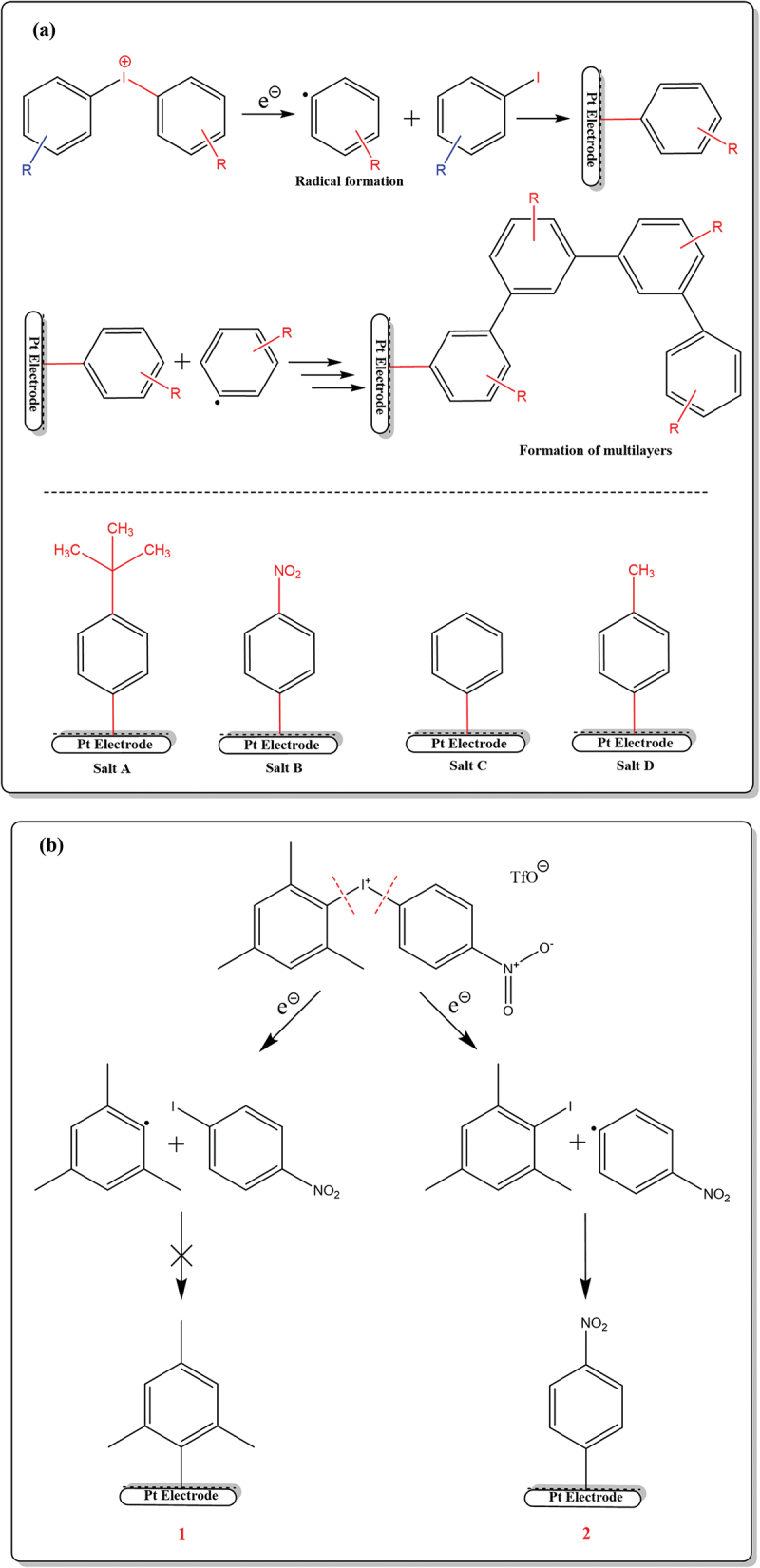
The general mechanism of electroreduction of iodonium salts, and the chemical structures of post-iodonium compounds grafted to the surface of Pt: (a) Mechanism of the formation of aryl radical (first step) and the attachment of the radical on the Pt electrode (second step) with further formation of multilayers with successive cycles (third step); (b) Mechanism of bond cleavage, aryl radical formation and attachment of the radical to the Pt electrode for Salt B. Mechanism pathway 2 shows the actual process taking place during the electroreduction.

Iodonium salts follow a similar one electron mechanism of electroreduction as diazonium salts [14] in which iodonium salt is reduced into the corresponding aryl radical attaching to the electrode surface and the charge stabilized counterpart of the molecule dissipates into the solution. Figure 2(a) shows the reaction mechanism followed by a schematic representation of organic compounds anchoring to the Pt surface after electrografting. As it can be seen in the diagram, half of the molecule is grafted onto the electrode surface, while the other half takes the form of the corresponding organic iodide. Ideally, an optimized process of electrochemical deposition of iodonium salts should provide an organic monolayer [7]. However, the reactivity of iodonium salts favors the formation of multilayers, particularly when more than 1 CV cycle is performed [14].

In the case of Salt B, which is an unsymmetrical molecule, the possibility of cleavage exists for both sides of the iodine cation, although only a part of the molecule with the nitro group in the *para* position can be deposited on the surface of the electrode due to steric hindrance [42,43]. It is important to mention that asymmetrical iodonium salts usually can cleavage for both possible radicals, which can react with surface. One of the ways to obtain high regioselectivity is grafting *via* a plasmon-assisted transformation [44]. Here, the regioselectivity is obtained due to the methyl groups attached in 2,4 and 6 position of phenyl ring in one of the part of molecule. The mechanism of attachment of aryl radical onto Pt electrode for Salt B is shown in Figure 2(b). Mechanism pathway 1 is unattainable, therefore the deposition of Salt B proceeds through mechanism pathway 2.

### 3.2. Electrogravimetric analysis

**Figure 3.**
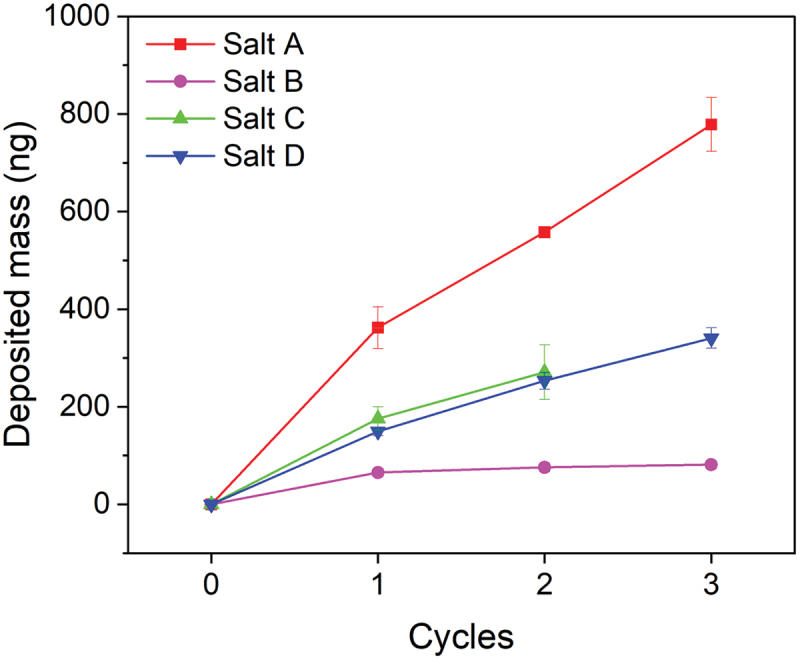
Change in mass deposited on the electrode depending on the number of reduction CV cycles for iodonium Salts A-D. Mass of the deposit resulting from the electroreduction of Salt C was recalculated to eliminate the effect of insoluble iodobenzene.

The electrografting process was studied with use of an electrochemical quartz crystal microbalance, which allowed to track changes in the mass of the working electrode as the function of applied potential (Figure 3). Salt A was deposited most efficiently (778.3 ± 55.1 ng), whereas Salt B, Salt C and Salt D were deposited in a significantly lower quantity (81.3 ± 7.8 ng for Salt B, 270.7 ± 56.1 ng for Salt C and 340.6 ± 20.8 ng for Salt D, respectively). The variability in deposited mass can be easily explained by different structures of radicals formed through the process of electrochemical reduction, and their easiness to attach to the surface. In the case of Salt C, the amount of deposited mass was corrected by subtracting the mass of iodobenzene, a side produce of the electroreduction process, which is insoluble in water [45] and is adsorbed on the surface of the electrode [46]. As shown in Figure 1, during the grafting process only half of the compounds react with the surface. The other half of the compounds forms an organoiodine derivative, i.e. 4-tertbutyliodobenzene, 2,4,6-trimethyliodobenzene, iodobenzene, or 4-iodotoluene for Salts A-D, respectively. Precipitation was not observed during the reduction of other salts, due to the solubility of 2,4,6-trimethyliodobenzene, iodobenzene and 4-tertbutyliodobenzene in acetonitrile [47]. The low deposited mass in the case of the reduction of Salt B can be explained by the deactivating character of a nitro group.

### 3.3. Surface properties

**Figure 4.**
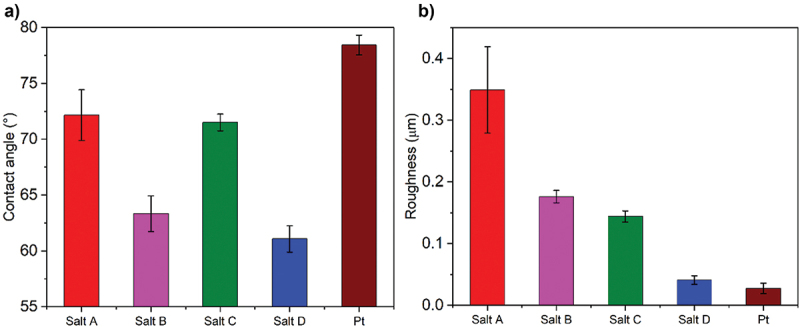
Wettability (a) and roughness (b) analysis of electrode surfaces modified with electrodeposited iodonium salts compared with a bare Pt electrode.

It is known that surface modification with a grafted organic layer may lead to a change in its wettability [48]. To verify this thesis, water contact angle of modified electrodes was measured (Figure 4(a)). In general, electrografting of all investigated iodonium salts led to a decrease in surface wettability compared to the unmodified Pt surface (θ = 78 ± 1°). The lowest value of contact angle was noted for Salt D (θ = 61 ± 1°), which does not differ significantly from a contact angle of a surface modified with Salt B (θ = 63 ± 2°). Values closer to unmodified Pt were found for both Salt A (θ = 72 ± 2°) and Salt C (θ = 72 ± 1°). In general, wettability depends on the homogeneity of the surface, substrate geometry, surface energy, surface roughness, etc [49]. Here, the difference in the contact angle of the modified electrodes and Pt is related to the substrate effect. For Salt A and Salt D, the presence of methyl group at terminal position influences the decrease in the contact angle, however the presence of a methyl group in Salt D significantly decreases the contact angle compared to the bulky group in Salt A. Even though the presence of nitro substituent is expected to lower the value of contact angle, the hydrophilic effect of this functional group is compensated by roughness of the surface, as discussed in the next sections [50].

All studied surfaces should be considered as moderately hydrophilic, making them suitable for the attachment of proteins, polymers, and polypeptides [31,51]. This effect was observed in our previous study using Pt/thermanox electrodes modified through the electroreduction of the diazonium layers and functionalized with polylysine [13]. Modification of the surface with a few nanometer thick adhesive layer of hydrophilic polymers (polyurethanes) was also introduced as an efficient method to achieve strong adhesion of different conducting polymers on various substrates, particularly under wet conditions [31]. In the light of the above, it is expected that moderate hydrophilicity of electrode’s surfaces modified with electrodeposited iodonium salts should promote the adhesion of proteins, cells, and polymers, and particularly a water-based PEDOT:PSS dispersion.

**Figure 5.**
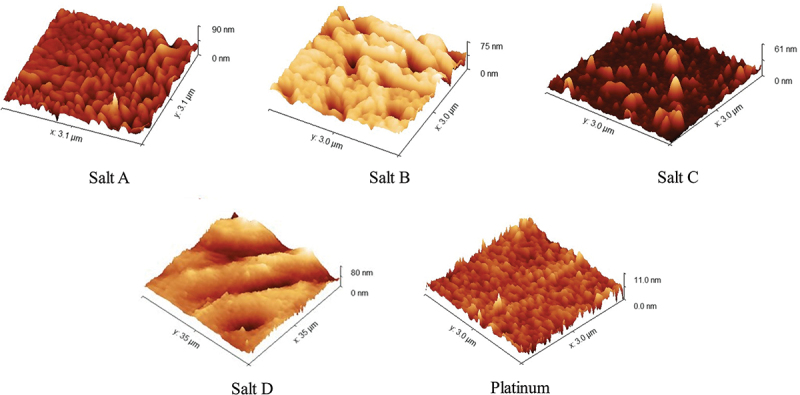
AFM images collected after the electrodeposition of the iodonium salts on the platinum electrode, compared with the pristine platinum substrate.

Surface roughness is another parameter that governs the adhesive nature of a substrate. Unmodified Pt surface was found to be rather flat, with S_a_ of 0.03 ± 0.01 µm. Roughness of the modified electrodes, assessed by means of an optical profilometry (Figure S1), increased substantially (Figure 4(b)), with the most significant change noted for Salt A (S_a_ = 0.35 ± 0.07 µm), and the least change noted for Salt D (S_a_ = 0.04 ± 0.01 µm). This provides a profound insight into the electrodeposition process, suggesting that due to the steric hindrance, the organic layer derived from Salt A (containing a bulky tert-butyl group) is not as compact as in the case of Salt D (containing a single methyl group). The results acquired by means of optical profilometry were supported by the AFM images (Figure 5), in which all modified surfaces were confirmed to possess more developed surfaces. In the case of Salt C, an irregular pattern of round structures may indicate precipitated iodobenzene, as described in the Section 3.2. Basing on the data, we estimate the thickness to be in the sub-micron range (Figure S2).

It is a well-known fact that adhesion is strictly related with surface roughness, and by modulating surface roughness, it is possible to form surfaces with either pro-adhesive or anti-adhesive properties [41,42]. Previous studies showed that rough surfaces can significantly facilitate the adhesion of polymers to metal substrates [52,53]. For instance, it was found that a rough Pt surface provides better adhesion of PEDOT compared to a smooth Pt surface [54]. Since after the modification of the Pt electrodes with iodonium salts surface roughness is increased for every investigated salt, we expect to observe better adhesion of polymers, and particularly PEDOT:PSS on the surface of modified electrodes.

### 3.4. Electrochemical properties

**Figure 6.**
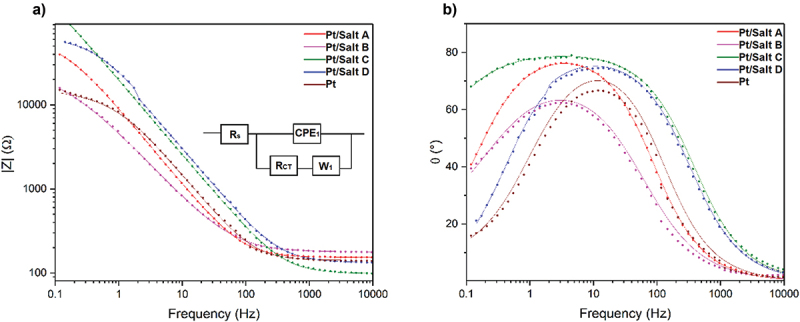
EIS data in the form of Bode plots of Pt electrodes modified with organic layers: impedance module vs. frequency (a), phase angle vs. frequency (b). Experimental data are presented as dots, while simulated spectra are presented as solid lines.

Electrochemical impedance spectroscopy was used to analyze the effect of the process of electrodeposition of iodonium salts on the electrochemical properties of Pt electrodes, including their conductivity and capacitance (Figure 6). Bode plots presenting the variation in the impedance module vs. frequency (Figure 6(a)) indicated a similar shape of the impedance profiles for all investigated samples, showing that the modification did not compromise the conductivity of a Pt electrode. Bode plots presenting the variation in the phase angle vs. frequency (Figure 6(b)) indicated the capacitive behavior of all investigated surfaces, with a single peak at the frequency between 1–10 Hz, attributed to a single time constant resulting from the presence of a single capacitive element in the equivalent electric circuit.

In an effort to describe the mechanism of charge transfer, modified Randles circuit was selected to represent the electrical behavior of coated electrodes. This circuit includes the following components: solution resistance (R_s_), electrode capacitance (CPE), charge transfer resistance (R_CT_), and a parameter related to the diffusion of charge (W). Experimental data were fitted to match the proposed circuit, and resulting values were presented in Table 1 and Figure 6(a,b) (solid lines). The difference between electrical parameters of a bare Pt electrode and Pt electrodes coated with electroreduced iodonium salts indicated successful surface modification. It was particularly visible in the case of R_CT_, which is a predictor of the efficiency of charge transfer between the electrolyte and the electrode. For example, for the electrode modified with Salt C, R_CT_ was equal to 451 ± 79 kΩ, and for the electrode modified with Salt D R_CT_ was equal to 62 ± 1 kΩ. These values were much higher than the R_CT_ for a bare Pt electrode (12.3 ± 0.7 kΩ), confirming the presence of the organic layer partially blocking the electron transfer on surface of the Pt electrode. The capacitive character of electrode modifications was confirmed by high values of n parameter, which approached 1 as in the case of the ideal capacitor. When comparing the double layer capacitance calculated from the constant phase element parameters according to the Equation (1), the highest capacitance was noted for Salt B (693.5 ± 0.1 µF) and the lowest capacitance was noted for Salt D (30.4 ± 0.2 µF), which was slightly lower than the capacitance of an unmodified Pt electrode (42.2 ± 0.1 µF).

**Table 1.** Summary of the electrical properties of electrodeposited samples derived from the equivalent circuit analysis; R_S_ – solution resistance, CPE – constant phase element, n,P – parameters of CPE, R_CT_ – charge transfer resistance, A_w_ – Warburg diffusion coefficient, χ^2^ – goodness of fit.

Samples	R_S_ (Ω)	n (CPE_1_)	P∙10^5^	R_CT_ (kΩ)	C (µF)	A_W_ (kΩ/s^1/2^)	χ^2^ (%)
Salt A	153 ± 1	0.911 ± 0.002	2.03 ± 0.02	56.1 ± 1.8	58.1 ± 0.1	2.572 ± 0.001	2.0
Salt B	178 ± 3	0.809 ± 0.006	4.61 ± 0.11	21.0 ± 1.9	693.5 ± 0.1	2.878 ± 0.002	5.2
Salt C	98 ± 1	0.895 ± 0.002	0.92 ± 0.01	450.9 ± 78.8	47.1 ± 0.2	67.461 ± 0.058	2.7
Salt D	133 ± 1	0.892 ± 0.002	0.82 ± 0.01	61.7 ± 1.4	30.4 ± 0.2	0.301 ± 0.001	2.3
Pt	141 ± 4	0.913 ± 0.007	1.62 ± 0.05	12.3 ± 0.7	42.2 ± 0.1	1.580 ± 0.001	6.9

### 3.5. Case study: performance of PEDOT:PSS coated on electrodeposited iodonium-based layers

**Figure 7.**
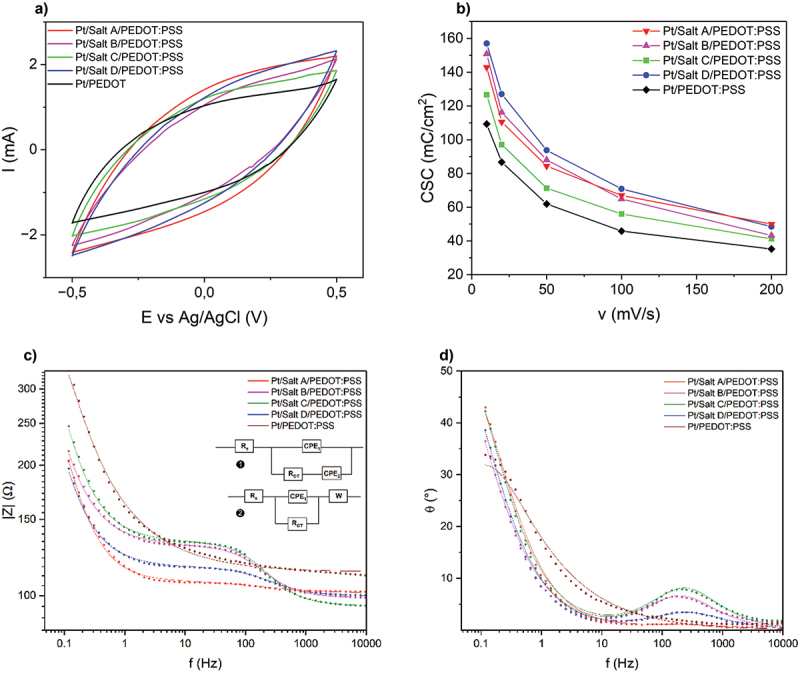
Electrochemical characterization of PEDOT:PSS deposited on Pt electrodes modified with organic layers: (a) CV curves; (b) charge storage capacities as a function of a scan rate; (c) impedance module vs. frequency Bode plot, with the circuits [1] Pt/Salt/PEDOT:PSS [2], Pt/PEDOT:PSS; (d) phase angle vs. frequency Bode plot. Experimental data are presented as dots, while simulated spectra are presented as solid lines.

As a case study used to verify the applicability of iodonium-derived organic layers, they were used to modify the surface of Pt electrode prior to the deposition of PEDOT:PSS. Since the presence of a pro-adhesive layer is expected to enhance charge transfer characteristics of the system, just as it was reported for diazonium-based coatings [20], the modified electrodes were investigated in terms of their electrochemical behavior. CV curves of all investigated samples (Figure 7(a)) show a shape typical for PEDOT:PSS, without clearly developed redox peaks [55]. Interestingly, the recorded currents were higher for PEDOT:PSS deposited on the surface of modified electrodes than observed for PEDOT:PSS deposited directly on the surface of a Pt electrode, suggesting the positive effect of the presence of organic layer on the capacitance of PEDOT:PSS. The ability of investigated materials to accumulate electric charge was quantified in terms of a charge storage capacity, CSC (Figure 7(b)), which is a parameter used to measure the amount of charge that can be reversibly stored in the electrode per unit geometric area [56], what is particularly important in designing biomedical electrodes and energy storage devices [40]. Interestingly, modification of Pt surface prior to the deposition of PEDOT:PSS layer led to the increase in the CSC values for all scan rates, with the most prominent effect for a surface modified with Salt D. For instance, at a scan rate of 100 mV/s, CSC for a surface of Pt/Salt D/PEDOT:PSS (70.9 mC/cm^2^) was 50% higher than the one calculated for a Pt/PEDOT:PSS surface (45.8 mC/cm^2^). The presence of Salt A and Salt B as the intermediate layer also led to higher CSC values, but with a lower extend than in the case of Salt D (CSC of 64.9  mC/cm^2^ and 67 mC/cm^2^ for Salt A and Salt B, respectively, at 100 mV/s).

EIS analysis was used to further evaluate electrical properties of PEDOT:PSS layers deposited on modified electrodes (Figure 7(c,d)). In contrast to the results presented in Figure 6(a), where the presence of organic layers resulted in a partial blocking of the electrode’s surface and the slight increase in the impedance module, the Bode plots of PEDOT:PSS coated electrodes (Figure 7(c)) indicated the decrease in the impedance profile, particularly in the case of electrodes modified with Salt A and Salt D. The presence of two capacitive peaks in the phase angle vs. frequency Bode plots (Figure 7(d)) suggests that the electrical equivalent circuit suitable for the description of charge transfer processed occurring in Salt/PEDOT:PSS coatings should contain two capacitive elements. Accordingly, the experimental EIS data was fitted in the circuit consisting of a solution resistance (R_S_) connected in series with the first constant phase element (CPE_1_) connected in parallel with a resistor (R_CT_) connected in series with a second constant phase element (CPE_2_). For a Pt/PEDOT:PSS sample, the experimental data was fitted in the circuit, in which the second constant phase element was replaced with the Warburg diffusion element. This behavior corresponds to the presence of a single layer of PEDOT:PSS, without the presence of an additional organic moiety, which is evident in the Bode plot showing only one capacitive peak. The accuracy of the used model was confirmed by a low value of a deviation between experimental and fitted data (<2%). The presence of two capacitive elements is easily explained by the presence of two layers on the surface of the electrode that can participate in charge accumulation, namely an electrodeposited organic layer and a drop-casted PEDOT:PSS layer. The addition of a second capacitive element manifests the dominant capacitive behavior within the Pt/Salt/PEDOT:PSS systems. Still, the capacitive character of PEDOT:PSS is not ideal (*n* < 1), hence the system should be understood as a pseudocapacitor, which is able to store charge both faradaically and through an electrical double layer [49].

While analyzing the parameters of the circuit (Table 2), it is clear that the presence of an organic deposit changes the conductivity of the electrode, what can be concluded from the values of the charge transfer resistance. After depositing PEDOT:PSS on the pretreated electrodes, charge transfer resistance is found to decrease from a kΩ range to Ω range, and more precisely to the values of 38.8 ± 0.9 Ω as noted for Pt/Salt C/PEDOT:PSS, 32.1 ± 0.8 Ω for Pt/Salt B/PEDOT:PSS, 16.8 ± 0.6 Ω for Pt/Salt D/PEDOT:PSS, and finally 8.1 ± 1.0 Ω for Pt/Salt A/PEDOT:PSS – all values lower than in the case of Pt/PEDOT:PSS without the presence of an organic layer (100 ± 8.9 Ω). The pre-exponential factor P associated with CPE_2_ also has a higher value for PEDOT:PSS coating on the surfaces modified with Salt B and Salt C compared to those modified with Salt A and Salt D, which shows that the capacitive behavior is also more dominant for Salt B and Salt C. This difference can be related to the functional groups present on the surface of the modified electrodes. The effect of electron donating (ED), electron withdrawing (EW) and bulky groups present on Pt surface can significantly impact the impedance of the surface. Nitro groups having the electron withdrawing effect decrease the electron density at Pt surface, which decreases the conductivity and increases the impedance as seen after coating the surface with PEDOT:PSS. However, electron donating groups (tert-butyl and methyl) increase the density of the electron on the surface enhancing the conductivity of the surface and decreasing the impedance [51]. This phenomenon can be observed in the high charge transfer resistance in case of Salt B and lower in Salt A and Salt D. ED and EW effects affect the adhesion of PEDOT:PSS on the surface and the impedance of the layers.

**Table 2.** Summary of the electrical properties of electrodeposited samples functionalized with PEDOT:PSS derived from the equivalent circuit analysis; R_S_ – solution resistance, R_CT_ – charge transfer resistance, CPE – constant phase element, n_1,2_,P_1,2_ – parameters of CPE_1,2_, χ^2^ – goodness of fit; for Pt/PEDOT; P_1_,n_1_ – parameters of CPE, Aw_1_ – Warburg diffusion coefficient.

Samples	R_s_ (Ω)	R_CT_ (Ω)	P_1_∙10^5^	n_1_	P_2_∙10^3^	n_2_	A_W_ (kΩ/s^1/2^)	χ^2^ (%)
Pt/Salt A/PEDOT:PSS	101.2 ± 0.4	8.1 ± 1.0	235.04 ± 0.18	0.58 ± 0.02	6.56 ± 0.17	0.88 ± 0.01	–	1.34
Pt/Salt B/PEDOT:PSS	98.9 ± 0.5	32.1 ± 0.8	6.81 ± 0.56	0.88 ± 0.01	9.54 ± 0.22	0.79 ± 0.01	–	1.35
Pt/Salt C/PEDOT:PSS	94.7 ± 0.6	38.8 ± 0.9	4.98 ± 0.38	0.87 ± 0.01	7.49 ± 0.18	0.82 ± 0.01	–	1.56
Pt/Salt D/PEDOT:PSS	99.8 ± 0.4	16.8 ± 0.6	13.64 ± 0.18	0.83 ± 0.01	10.07 ± 0.02	0.81 ± 0.01	–	0.38
Pt/PEDOT:PSS	113.4 ± 0.7	100 ± 8.9	0.02 ± 0.01	1.00 ± 0.01	–	–	96.6 ± 2.6	1.18

**Figure 8.**
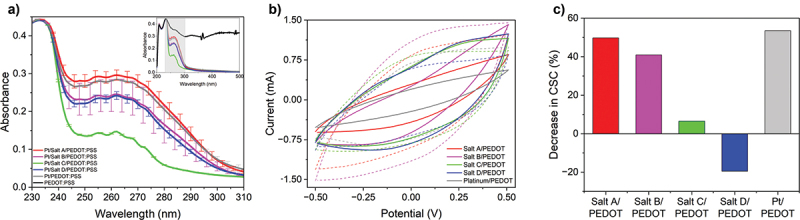
(a) UV-Vis spectra of PBS solution after 30 days of ageing in the spectral region from 230 nm to 310 nm; inset: UV-Vis spectra of PEDOT:PSS in the range from 220 nm to 500 nm; (b) CV curves for samples after ageing (solid lines) and before (dashed lines) at scan rate = 100 mV/s; (c) Decrease in CSC values at scan rate = 100 mV/s for samples after 30 days of ageing test.

Apart from the effect of organic layers on the charge transfer mechanism, the presence of such layers could also affect the adhesion of PEDOT:PSS, as shown in our previous study [20]. To assess stability of PEDOT:PSS coatings deposited on the surface of iodonium-derived organic layers ageing test and tape test were performed. For an ageing test, the samples were subjected to a 30-day immersion in PBS, and the disintegration of the polymer layer was evaluated by analyzing traces of PEDOT (characteristic peak around 325 nm) and PSS (characteristic peaks at 250–270 nm) in the solution using UV-Vis spectroscopy (Figure 8(a)). Consequently, a substantial decrease in the absorbance in the region assigned to PSS was noted for the modified electrodes (except for Pt/Salt A/PEDOT:PSS), stating that these modifications increased the integrity of PEDOT:PSS coating by 16.6% for Pt/Salt D/PEDOT:PSS and 53.4% for Pt/Salt C/PEDOT:PSS (both values estimated basing on the absorbance at 268 nm), by eliminating the spontaneous elution of a doping agent, PSS. A large standard deviation between technical replicas of Pt/SaltB/PEDOT:PSS could suggest the inhomogeneity of the electrode after its modification with iodonium salts, leaving some uncoated areas that decreases its overall performance. In comparison with diazonium salts, iodonium salts showed a stronger effect on stability of PEDOT:PSS. In our previous article [20], the decrease in absorbance at 268 nm was lower than 2%, stating that the presence of diazonium moiety could only slightly decrease the rate at which PSS elutes into the solution.

The analysis of an electrochemical performance in terms of CSC change after subjecting the modified electrodes to ageing studies revealed the beneficial effect of the presence of iodonium-derived layers on the capacitance of PEDOT:PSS (Figure 8(b,c)). The highest decrease in CSC was observed for an unmodified Pt/PEDOT:PSS electrode (53.5%) highlighting its instability. The presence of an organic layer, particularly those derived from the reduction of Salt C and Salt D, was found to substantially reduce the loss in CSC (Figure 8(c)), which was in line with the results acquired from UV-Vis stability studies. Interestingly, the capacitance of Pt/Salt D/PEDOT:PSS was found to improve by 20% as a result of ageing, which should be related to the changes in microstructural crystallinity and morphology upon immersion in an aqueous solution, as described earlier [57,58]. An additional insight into the aqueous stability of PEDOT:PSS layers was provided by the results of a tape test (Figure S3). The visual inspection of surfaces after delamination indicated the beneficial effect of the presence of organic layers on the stability of a PEDOT:PSS film, with the exception of the surface modified with Salt C, where the delamination was observed exactly in the place where the electrode was immersed during the electrografting process, indicating the weak adhesive properties of this salt.

**Figure 9.**
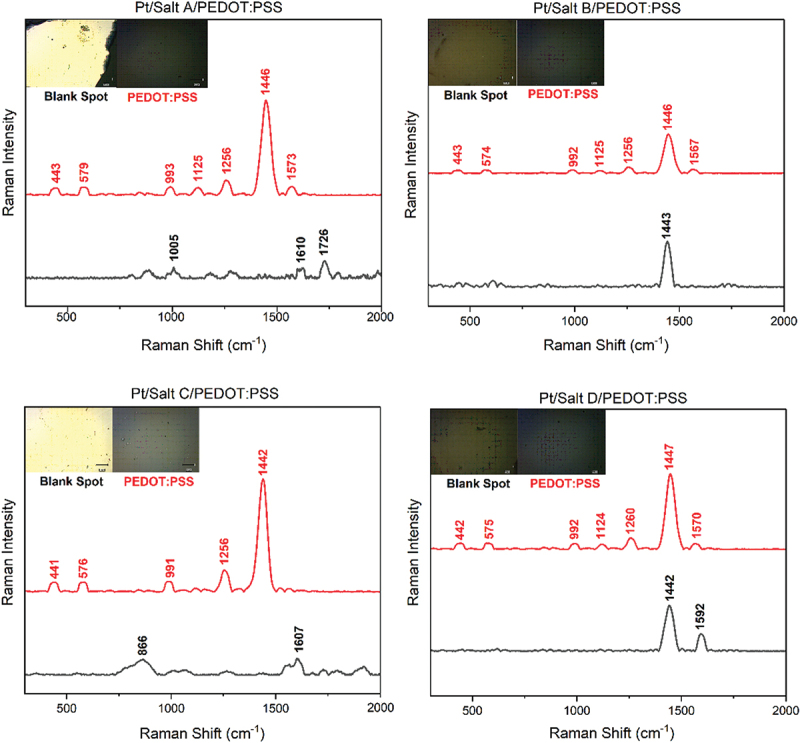
Raman spectra of Pt/Salt/PEDOT:PSS electrodes after a tape test; red spectra represent the electrodes before a tape test, and black spectra represent the electrodes after a tape test with the inset showing the images of the area of the experiment.

Raman spectra were collected to monitor the chemical composition of the electrodes before and after a tape test (Figure 9). The results showed the characteristic peaks for PEDOT:PSS at 1573 cm^−1^ (PEDOT C_α_=C_β_), 1446 cm^−1^ (PEDOT C_α_=C_β_), 1256 cm^−1^ (PEDOT C_α_=C_α`_), 993 cm^−1^ (PSS), 579 cm^−1^ (PEDOT C-O), which were present in every sample (with a negligible peak shift) before the tape test [59,60]. Interestingly, Raman spectra collected on the surface of the electrodes after delamination also showed some peaks corresponding to the PEDOT:PSS (particularly for Salt B and Salt D), suggesting that a thin layer of PEDOT:PSS was still present on the surface.

## 4. Conclusion

In this study, we have verified the applicability of the process of electrochemical reduction of iodonium salts as way to form pro-adhesive coatings for the further deposition of PEDOT:PSS. Among investigated salts, it was bis(4-methylphenyl)iodonium hexafluorophosphate (Salt D) that produced organic layers able to increase the electrochemical performance of PEDOT:PSS in terms of high charge storage capacity (70.9 mC/cm^2^ at 100 mV/s) and low charge transfer resistance (16.8 ± 0.6 Ω), as well as increase its stability, most probably because of its most hydrophilic character among investigated surfaces (θ = 61 ± 1°). By the possibility to undergo the changes in microstructural crystallinity and morphology upon immersion in an aqueous solution, PEDOT:PSS deposited on the electrode modified with Salt D showed an increase in the capacitance as a result of ageing.

Our research proves that iodonium salts can be successfully treated as substituents for diazonium salts in the processes of surface modification. Such a conversion is particularly important in applications where spontaneous reduction is undesirable, e.g. pattering, but the need to use more negative potentials must be considered. Selected iodonium salts may be successfully used as ‘molecular glues’, increasing the adhesion of conducting polymers simultaneously improving their electrochemical properties. Since conducting polymer-coated electrodes are widely used in a variety of applications, from biomedical engineering to energy storage devices, the results of our studies are expected to further improve these fields by providing materials with enhanced stability and performance.

## Supplementary Material

Supplemental Material
