# Supplementary material for: Iodonium-based pro-adhesive layers for robust adhesion of PEDOT:PSS to surfaces
Source: Sci Technol Adv Mater. 2024 Apr 4;25(1):2338786. doi: 10.1080/14686996.2024.2338786 (PMC11047218; doi:10.1080/14686996.2024.2338786)
Supplement: Supplemental Material [file TSTA_A_2338786_SM6358.pdf]

Supplementary information: Iodonium-based pro-adhesive layers for robust adhesion of PEDOT:PSS to surfaces

Szymon Smołka[a], Taral Patel[a,b], Sandra Pluczyk-Małek[a,c], Roman Turczyn[a,c], Katarzyna Krukiewicz[a,c,*]

[a]*Department of Physical Chemistry and Technology of Polymers, Silesian University of Technology, M. Strzody 9, 44-100 Gliwice, Poland*

[b]*Joint Doctoral School, Silesian University of Technology, Akademicka 2A, 44-100 Gliwice, Poland*

[c]*Centre for Organic and Nanohybrid Electronics, Silesian University of Technology, Konarskiego 22B, 44-100 Gliwice, Poland*

* Correspondence: Katarzyna Krukiewicz, katarzyna.krukiewicz@polsl.pl

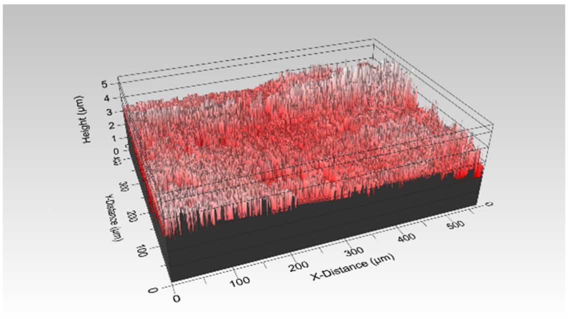

Salt A

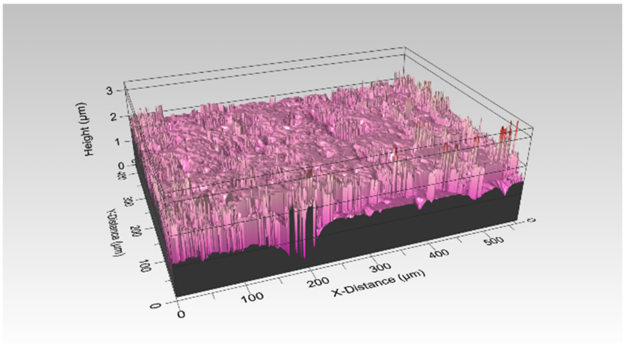

Salt B

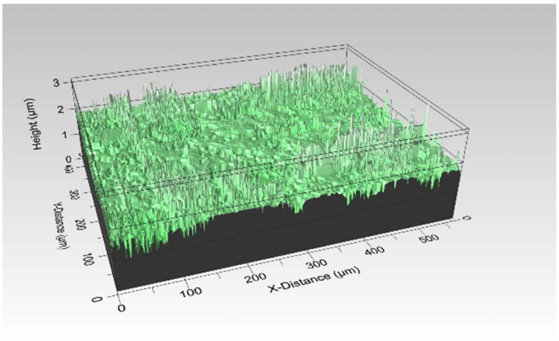

Salt C

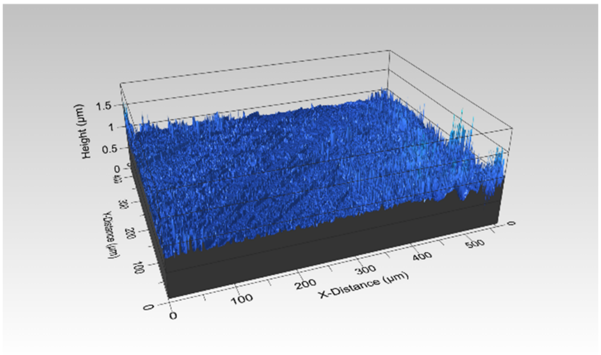

Salt D

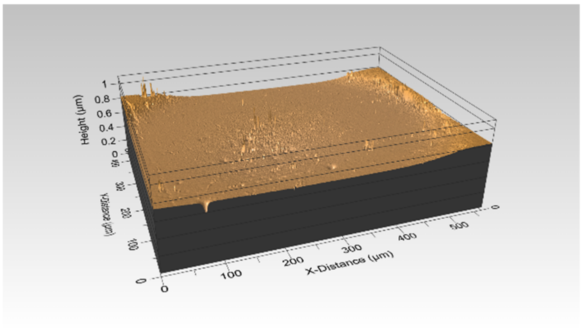

Platinum

Figure S1. Optical images collected after the electrodeposition of the iodonium salts on the platinum electrode, compared with the pristine platinum substrate.

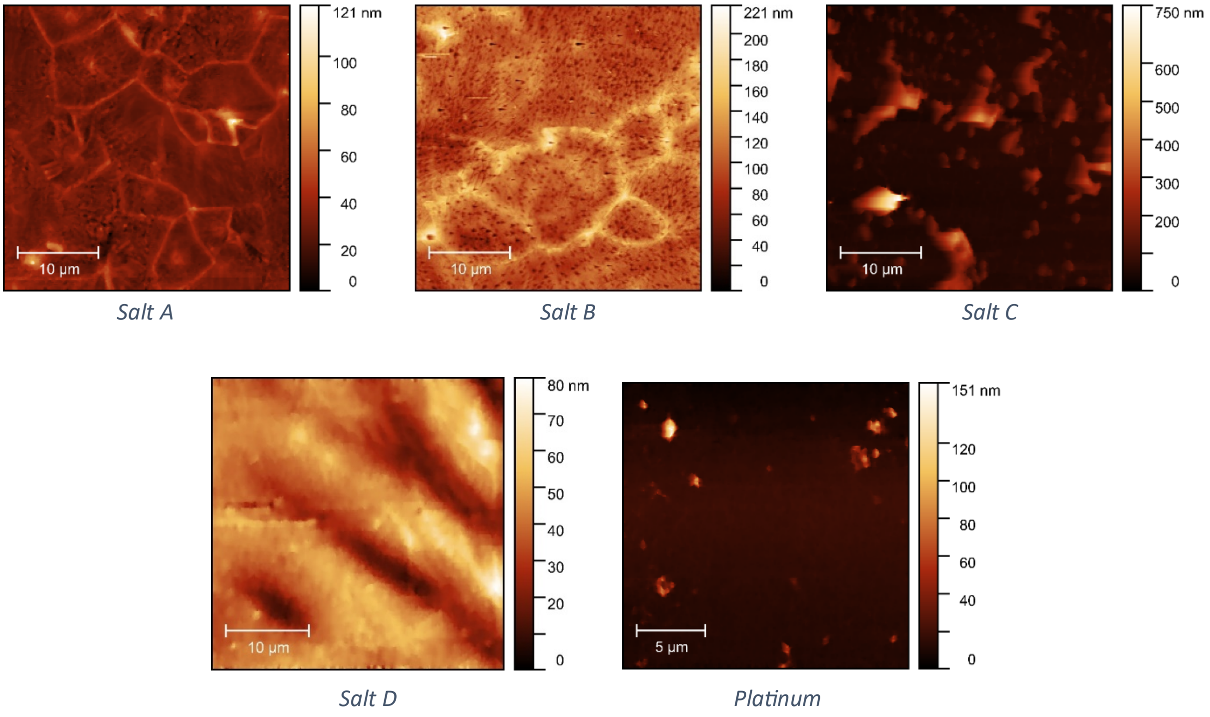

Figure S2. AFM images collected after the electrodeposition of the iodonium salts on the platinum electrode, compared with the pristine platinum substrate.

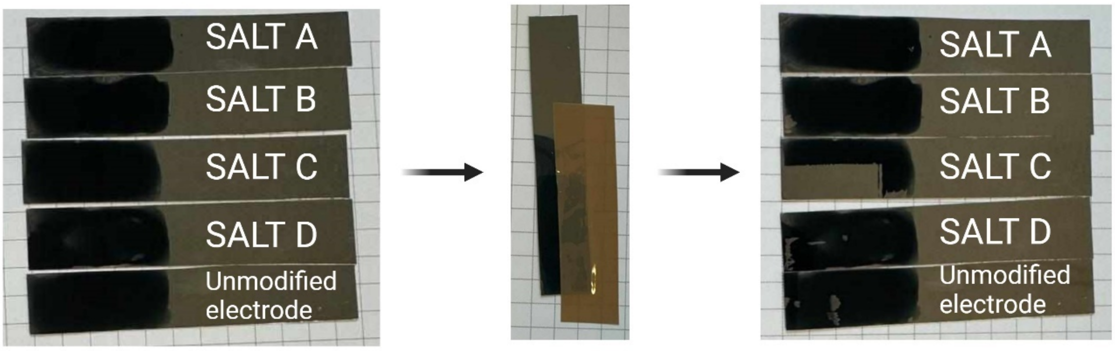

Figure S3. Photos of electrodes covered by PEDOT:PSS before (on left) and after (on right) an adhesive tape test.
